# Impacts of Surface Hydrophilicity of Carboxylated Polyethersulfone Supports on the Characteristics and Permselectivity of PA-TFC Nanofiltration Membranes

**DOI:** 10.3390/nano11102470

**Published:** 2021-09-22

**Authors:** Yingfu Lian, Gang Zhang, Xiaojun Wang, Jie Yang

**Affiliations:** 1 College of Polymer Materials Science and Engineering, Sichuan University, Chengdu 610064, China; laotan85@126.com; 2 Analytical & Testing Center, Institute of Materials Science and Technology, Sichuan University, Chengdu 610064, China; wangxj@scu.edu.cn; 3 State Key Laboratory of Polymer Materials Engineering of China, Sichuan University, Chengdu 610064, China

**Keywords:** carboxylated PES copolymer, support effect, interfacial polymerization, selective PA layer, TFC nanofiltration membrane

## Abstract

Our current study experimentally evaluates the impacts of surface hydrophilicity of supports on the properties of polyamide (PA) thin-film composite (TFC) nanofiltration (NF) membranes. A series of “carboxylated polyethersulfone” (CPES) copolymers with an increasing “molar ratio” (MR) of carboxyl units were used to prepare supports with diverse surface hydrophilicities by the classical nonsolvent-induced phase separation (NIPS) method. Then, the PA-TFC NF membranes were finely fabricated atop these supports by conventional interfacial polymerization (IP) reactions. The linkages between the surface hydrophilicity of the supports and the characteristics of the interfacially polymerized PA layers as well as the permselectivity of NF membranes were investigated systematically. The morphological details of the NF membranes indicate that the growth of PA layers can be adjusted through increasing the surface hydrophilicity of the supports. Moreover, the separation results reveal that the NF membrane fabricated on the relatively hydrophobic support exhibits lower permeability (7.04 L·m^−2^·h^−1^·bar^−1^) and higher selectivity (89.94%) than those of the ones prepared on the hydrophilic supports (14.64~18.99 L·m^−2^·h^−1^·bar^−1^ and 66.98~73.48%). A three-stage conceptual scenario is proposed to illustrate the formation mechanism of the PA layer in NF membranes, which is due to the variation of surface hydrophilicity of the supports. The overall findings specify how the surface hydrophilicity of the supports influences the formation of PA layers, which ultimately defines the separation performances of the corresponding NF membranes.

## 1. Introduction 

Currently, the most predominant nanofiltration (NF) membranes and reverse osmosis (RO) membranes are prepared as polyamide thin-film composite (PA-TFC) membranes to produce freshwater from seawater and brackish groundwater [1,2,3]. A classic PA-TFC membrane is composed of three parts: a top ultrathin selective PA layer, a middle porous support layer and a bottom nonwoven fabric layer [4,5]. Not only is the unique structure of the PA-TFC membrane beneficial in reducing the fluid mass transfer resistance through it, but it is also helpful in independently tailoring each layer of it to achieve the best overall membrane performance.

From the lab bench to the market scale, the PA layer of TFC membranes is typically synthesized via the interfacial polymerization (IP) of diamine derivatives in the aqueous phase and trifunctional acyl chloride in the organic phase on the porous support membrane [6]. During the fabrication process, for the solubility of amine derivatives in the organic phase to always be higher than that of acyl chloride in the aqueous phase, the IP reaction primarily takes place on the organic side [7]. Favorably, when the IP reaction is slowed down due to self-termination, a cross-linked PA layer with a finite thickness is formed. It has long been commonly considered that the performance of a TFC membrane is decided largely by its selective PA layer, and the porous support layer and the nonwoven fabric layer are merely mechanically robust substructures. Based on this consideration, in the last three decades, tremendous efforts have been made in studies about how to control the IP process to develop high-performance PA-TFC membranes, involving the development of novel monomers [8], solvents [9] and additives [10,11], the adjustment of polymerization and curing conditions [12,13] etc.

Recently, some studies have demonstrated that surface porous characteristics (e.g., pore size [14,15,16] and porosity [17]) and other surface properties (e.g., roughness [18] and hydrophilicity [19]) of supports also play substantial roles in the formation of selective PA layers and, hence, the performance of TFC membranes. Significantly, Ghosh et al. [19] found that the TFC membranes fabricated on supports with similar surface hydrophilicity and roughness, but diverse surface pore sizes (ranged from 60 nm to 140 nm), showed almost identical performance. That is, the surface hydrophilicity and roughness have a greater effect in comparison to the surface pore sizes. Furthermore, by carrying out the interface polymerization process atop patterned supports, Maruf et al. [18] found that the growth of PA layers was non-conformal. Consequently, the most influential surface hydrophilicity becomes the focus for further research quite naturally.

Unfortunately, to date (to our knowledge), a general and reliable understanding regarding the effects of surface hydrophilicity of supports drawn from independent research is still being debated. For example, Ghosh et al. [19] suggested that a more permeable TFC membrane was produced on a support with higher hydrophobicity. However, Kim et al. [20] described that the water permeability of TFC membranes was enhanced with the increase in the surface hydrophilicity of supports. Thus, more convincing results about how the surface hydrophilicity of supports impacts the formation of the PA layer as well as the performance of TFC membranes are crucial for us.

**Figure 1:**
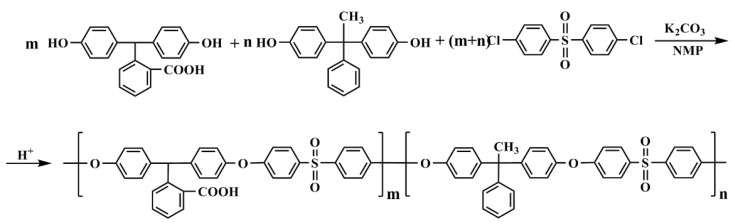
Synthesis routes of the carboxylated PES copolymers.

In view of this, through the preparation of NF membranes on our self-made supports by an IP reaction, the influences of the surface hydrophilicity of supports on the properties of TFC membranes are investigated emphatically in what follows. Generally, supports with a wide variety of characteristics may be obtained via three different approaches: (i) bulk modification of the support materials (pre-modification) [21], (ii) blending of the support materials with additives [22] and (iii) surface modification of the prepared supports (post-modification) [20]. The above methods have their rationality, but their well-known flaws also exist. For the bulk modification, most of the materials (e.g., polysulfone (PSF) or polyethersulfone (PES)) are commercial products, invariably leading to uncertainty about their chemical structure and components. Therefore, it is difficult to control the modification precisely. For the blend modification, the incorporated additives (e.g., polyethylene glycol (PEG) or polyvinyl pyrrolidone (PVP)) can be easily detached, resulting in the unstable properties of supports and, further, unreliable conclusions. For the surface modification, the surface characteristics of supports after processing are complicated and fluctuate. So, there is a great need to develop new materials that can be directly used to produce supports with finely tuned characteristics. As schematically illustrated in Figure 1, very recently, our group [23] successfully synthesized a series of PES with different contents of carboxylic acid groups in the pendant benzene ring (i.e., carboxylated PES (CPES)) via a copolymerization method. It was elucidated that these hydrophilic-modified PES copolymers did not show a substantial increase in swelling degrees by water, thus the mechanical instability. Moreover, the NF membranes fabricated with these copolymers by the classical NIPS method exhibited wide variations in antifouling performance due to their different surface hydrophilicities. Hence, CPES copolymers are ideal materials to reveal the connection between the properties of supports and the performances of NF membranes.

Based on the synthesis of a series of CPES copolymers with a different “molar ratio” (MR) of carboxyl units in the total molecule units (changed from 0% to 100%), which can be calculated via Equation (1), the supports were fabricated by the same NIPS method. Herein, the effects of the MR of carboxyl units in the CPES copolymers on the morphology, surface properties and separation performances of supports were studied in detail. Then, the NF membranes were prepared through IP on the above supports. The characteristics of the PA layers and the permselectivity of the corresponding TFC membranes were investigated thoroughly to shed light on the role of the surface hydrophilicity of the supports. Furthermore, the likely formation mechanism of the PA layers affected by the surface hydrophilicity of the supports during the IP process was elucidated. What needs to be pointed out is that the optimized parametric studies (e.g., monomer concentration, temperature and time of reaction and curing) of PA layers during the IP reaction are not reported in this paper.

(1) $$\mathrm{MR}=\frac{m}{m+n}\;\times \;100\%$$

## 2. Materials and Methods

### 2.1. Materials and Reagents

As shown in Figure 1, the CPES copolymers were synthesized through the method reported previously [23]. You may refer to our previous paper [23] for more information on CPES copolymers. Reagent grade materials, including N-methyl pyrrolidone (NMP), PEG with a weight averaged molecular weight of 20,000 g/mol, anhydrous piperazine (PIP, >99%), trimesoyl chloride (TMC, >98%), n-hexane (>99%), magnesium sulfate (MgSO_4_) and hydrochloric acid (HCl) along with sodium hydroxide (NaOH), were purchased from Aladdin Reagent Co. Ltd. (Shanghai, China) and used as received without any further purification. Commercial polyester (PET) nonwoven fabrics with an areal density of 52.7 g/m^2^ were provided by Teijin Furuite Co. Ltd. (Osaka, Japan) and preserved in a warehouse with constant temperature (25 °C) and humidity (<20%). Unless mentioned otherwise, laboratory-prepared deionized water with conductivity <1 μS/cm was used in all experiments.

### 2.2. Preparation of Supports and Synthesis of PA-TFC NF Membranes

The supports were prepared by the NIPS method. Firstly, according to the concentration of the casting solutions (21 wt%), certain amounts of CPES copolymers with a different MR of carboxyl units and NMP were accurately weighed and then added to airtight glass bottles. After being stirred at 60 °C for 12 h, homogeneous casting solutions were obtained, which needed to be left still until all air bubbles disappeared before use. Next, the casting solutions were spread on the PET nonwoven fabrics with the initial film thickness of 230 μm using fully automatic equipment, and, subsequently, the casting films were immediately immersed into a coagulating bath (deionized water) at 15 °C to induce the phase inversion process. Finally, the resultant supports were washed thoroughly with deionized water and later stored in a refrigerator maintained at 4 °C.

The NF membranes were synthesized on the top of the supports prepared previously via the IP process. Initially, the supports taped to the glass plates were impregnated with an aqueous solution of 0.2% (*w*/*v*) PIP at 25 °C for 3 min. Then, the wetted supports were taken out and subjected to the air (RH: 50~60%), and the excess PIP solution was removed from the surface of the supports using a clean rubber roller. After that, the PIP-soaked supports were brought into contact with a hexane solution of 0.1% (*w*/*v*) TMC at 25 °C for 1 min, leading to the formation of ultrathin PA layers over the supports. Moreover, after their exposure to the hexane–TMC solution, the membranes were immediately placed in an oven at 75 °C for 4 min. Ultimately, the resulting NF membranes were washed thoroughly with deionized water and stored in a laboratory refrigerator maintained at 4 °C prior to use.

For the sake of brevity, hereinafter, the notations presented in Table 1 are used to denote the CPES copolymers, supports and NF membranes.

### 2.3. Characterization of Supports and PA-TFC NF Membranes

#### 2.3.1. Thickness of Support

The “thickness of nonwoven fabrics” (δ_n_) and the “total thickness of support and nonwoven fabrics” (δ_t_) were measured using an oil-proof digital display thickness gauge (Zhejiang Deqing Shengtaixin Electronic Technology Co. Ltd., Hangzhou, China) at eight different locations for each sample, and their average values were taken as the final result. Consequently, the “thickness of support” (δ_S_) can be computed through Equation (2).

(2) $$\delta_{S}{=\delta }_{t}-\delta_{n}$$

#### 2.3.2. Scanning Electron Microscope (SEM)

After being dried at 40 °C for 24 h, the surface morphology of all the membranes, including the supports and the NF membranes, was visualized by a JSM-7500F field-emission scanning electron microscopy (FESEM) (Japan Jeol Company, Tokyo, Japan). To observe the cross-section morphologies, the wet samples needed to be freeze fractured in liquid nitrogen before drying. Then, an Apreo S SEM (Thermo Fisher Scientific, Waltham, MA, USA) was adopted to visualize the cross-section morphology of the supports, and a JSM-7500F FESEM was used to analyze the cross-section morphology of the PA layer in the TFC NF membranes.

#### 2.3.3. Atomic Force Microscope (AFM)

A quantitative surface roughness analysis of all the membranes was estimated by a Smart SPM AFM (AIST-NT). For each test run, a 5 × 5 μm^2^ topographical surface image of the dried samples was scanned using the tapping mode in air. The measured results were reported in terms of root mean square (RMS, i.e., the standard deviation in height values) roughness. At least five random regions were scanned for each sample to obtain the final average roughness result.

#### 2.3.4. Contact Angle (CA)

The relative surface hydrophilicity of all the membranes was evaluated by the sessile drop method using a DSA10 contact angle measuring instrument (KRüSS GmbH, Hamburg, Germany). At least five valid equilibrium contact angles defined by the average of the right and left contact angles of the deionized water on each dried surface of the samples were obtained. Finally, the average contact angle and the standard deviation of the membranes were calculated using the above test data.

#### 2.3.5. X-ray Photoelectron Spectrometer (XPS)

To reveal the surface structure of the PA layer in the TFC NF membranes, the detailed surface elemental information of the PA layers was assessed by a Kratos AXIS Ultra XPS (Shimadzu, Columbia, MD, USA) employing a monochromatized Al-Kα X-ray source (1.49 keV). Then, the Casa XPS software was adopted to perform the XPS analyses. (Note: the binding energy offset was corrected based on the binding energy of adsorbed carbon at 284.6 eV.)

### 2.4. Performance Evaluation of Supports and PA-TFC NF Membranes

**Figure 2:**
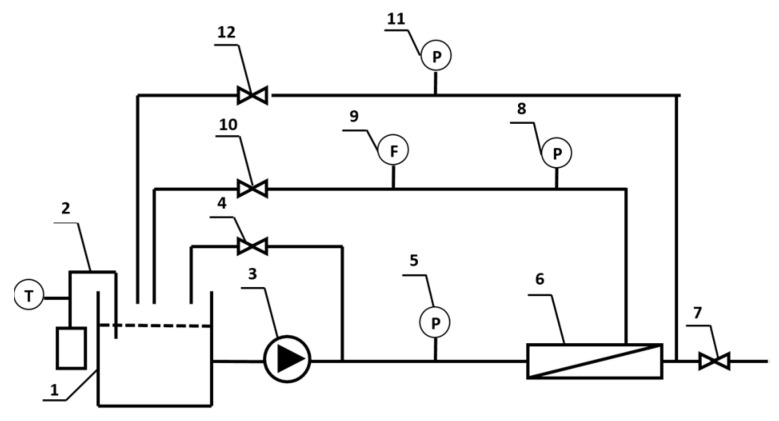
Schematic diagram of the set up for evaluating separation performances of membranes. 1: storage tank; 2: temperature control system; 3: booster pump; 4, 7, 10, 12: globe valve; 5, 8, 11: pressure gauge; 6: membrane test cell; 9: flow meter.

The “pure water permeability” (PWP) and “rejection rate” (R) of the supports were evaluated via the standard permeation test with a lab-built filtration apparatus as illustrated in Figure 2 (the effective filtration area is 7.0 × 10^−3^ m^2^). The dead-end filtration mode was adopted to measure the PWP. After the temperature of the deionized water in the storage tank was adjusted to 25.0 °C, the inlet pressure was slowly boosted to 0.150 ± 0.005 MPa along with a pre-pressure of 30 min. After that, the pressure was dropped to 0.100 ± 0.005 MPa, and then the test was run for 10 min in a steady state. Finally, we recorded the permeated volume of water that was collected in a small cylinder over 10 min since the beginning of the test. Equation (3) is the calculation formula of PWP.

(3) $$\mathrm{PWP}=\frac{V}{S\;\times \;\Delta P\;\times \;\Delta t}$$

where V (L) is the volume of water permeation collected within a time period of Δt (h), ΔP (bar) is the externally applied hydraulic pressure, and S (m^2^) is the effective filtration area. Note: the end result of PWP for each support is the average of five test runs.

To lessen the impact of concentration polarization, the cross-flow filtration mode was selected to obtain the rejection rate. Once the aqueous PEG solution (1000 mgL^−1^) was pumped into the storage tank and maintained at 25 ± 0.5 °C, the inlet pressure was slowly adjusted to 0.10 MPa. After stabilizing for 10 min, both the feed solution and the permeate solution were collected and later assayed by a LiquiTOC/TNb total organic carbon (TOC) analyzer (Elementar Analysen Systeme GMBH Co. Ltd., Langenselbold, Germany) to obtain the concentration values. The following Equation (4) was employed to calculate the rejection rates:

(4) $$R=\left(1\;-\;\frac{C_{p}}{C_{f}}\right)\times \;100\%$$

where C_p_ (mgL^−1^) is the calculated concentration of PEG in the permeate solution, and C_f_ (mgL^−1^) is the average of the initial concentration and the final concentration of PEG in the feed solution. Note: the final result of rejection rate for each support is the average of five test runs.

The above filtration apparatus was also used to evaluate the separation performances of the PA-TFC NF membranes. After starting the booster pump, the globe valves were slowly adjusted to meet the test conditions listed in Table 2. Through a 60 min steady operation, the sample solutions were collected and then used to conduct related tests. The above measurement was repeated three times and the average value was taken as the final result. The “water permeability” (A) of the NF membranes was also calculated from Equation (3). The conductivity of the permeate solution and the feed solution was measured by a DDS-307 conductivity meter (Shanghai Leici Instrument Co. Ltd., Shanghai, China). Thus, the “salt rejection” (R) of the NF membranes can be determined from Equation (5),

(5) $$R=\left(1\;-\;\frac{k_{p}}{k_{f}}\right)\;\times \;100\%$$

where k_p_ (μScm^−1^) is the measured conductivity of the collected permeate solution, and k_f_ (μScm^−1^) is the arithmetic mean of the initial conductivity and the final conductivity of the feed solution.

## 3. Results and Discussion

### 3.1. Effects of MR of Carboxyl Units in CPES Copolymers on the Properties of Supports

**Figure 3:**
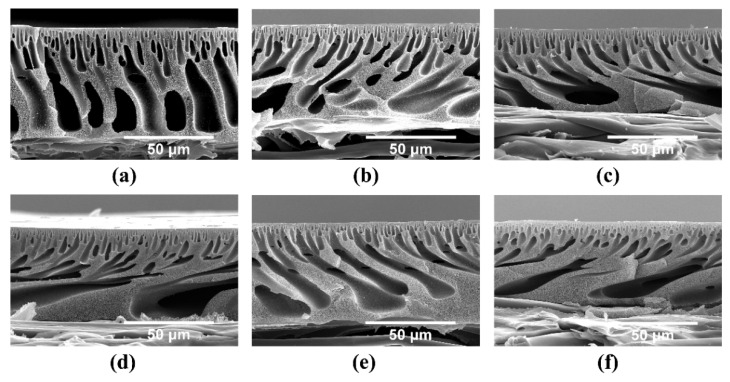
Overall cross-section SEM image of the supports: (**a**) S-0, (**b**) S-20, (**c**) S-40, (**d**) S-60, (**e**) S-80 and (**f**) S-100.

A series of supports (S-0~S-100) were fabricated with CPES copolymers by adopting the NIPS method. From the overall cross-section SEM images in Figure 3, the typical asymmetric structure (consisting of two layers, that is, a thin and dense top layer and a much thicker, porous sublayer) of the supports is still maintained with the increasing MR of carboxyl units in the CPES copolymers. The SEM observations illustrate that the precipitation type of phase inversion processes instantaneously demixes. Moreover, due to the tendency of carboxylic acids to “self-associate” via hydrogen bondings [24], the increase in the MR of carboxyl units in CPES copolymers can cause both intra- and inter-molecular entanglements and/or aggregations of polymer chains in the casting solutions. What follows is the increase in the viscosity of the casting solutions (S-0~S-100) from 1600 mPa·s to 4667 mPa·s (see Table S1, Supplementary Materials), which hinders the diffusional exchange rate between the NMP (solvent) and the water (nonsolvent). As such, the “angle effect” on the exchange between solvent and nonsolvent becomes increasingly more pronounced, hence leading to changes in the pores’ orientation (note: when fully automatic equipment was used to fabricate the supports in this study, the casting films went through the dynamic process of phase inversion in the coagulation bath). Moreover, the macrovoids gradually become increasingly bigger with the continual renewal of the polymer-poor nucleus boundary from S-0 to S-100.

The surface pore morphology measurement of the supports was performed with FESEM (×50,000, ×100,000 and ×150,000 magnification). However, as the surface pores were easily covered by the gold nanoparticles sprayed during the sample preparation process, it would be unrealistic to measure and calculate the pore characteristics from the surface FESEM images. Here, we used the molecular weight cut off (MWCO) (see Figure S1 and Table S2, Supplementary Materials) to estimate the surface pore size (d) of the supports. According to the relationship between MWCO and pore size proposed by Lentsch et al. [25] as shown in Equation (6), the surface pore size values are determined as listed in Table 3. It is worth pointing out that the surface pore size of the supports (range of 4~9 nm) is much smaller than that of a large majority of the previous ones. Moreover, hydrophilicity has a relatively greater impact compared to the surface pore size [19]. As such, it appears reasonable to ignore the influences of surface porous characteristics of the supports on the properties of the NF membranes.

(6) $$d=0.09\;\times \;{\left(MWCO\right)}^{0.44}$$

Other characteristic results, such as the thickness (δ_S_), roughness (RMS) and hydrophilicity (CA) of the supports, are also summarized in Table 3. As presented in this table, the average thickness of the supports is in the range of 48~50 μm, illustrating that the thicknesses have an inconspicuous dependence on the MR of carboxyl units in CPES copolymers.

The RMS roughness values increase slightly from 18.3 nm to 49.4 nm as the MR of carboxyl units in CPES copolymers increase. From previous studies [26,27], the variation of sizes of aggregated particles (or nodules), such as polymer aggregates or agglomerates of the polymer aggregates on the surface of the supports, is supposed to be the reason for the change in surface roughness. However, as the growth of the PA layer on the support is non-conformal [18], here, the effects of the roughness of the supports on the NF membranes are negligible.

With the increase in the MR of the carboxyl units in the CPES copolymers, the surface contact angle of the supports reduces from 90.43° for S-0 to 82.12° for S-100 (details about the data can be seen in Table S3 (Supplementary Materials)). Herein, it is remarkable that different from the “intrinsic contact angle” (θ_I_) of a material, the “apparent contact angle” (θ_A_) of a porous surface (e.g., porous membrane) obtained through the static sessile drop method, as described in Section 2.3.4, is also affected by its “surface roughness coefficient” (ψ) and “surface porosity coefficient” (f) as shown in Equation (7) [28]. Thus, as the CPES copolymers are hydrophilic (i.e., θ_I_ < 90°, see Table S4, Supplementary Materials), the surface hydrophilicity of the supports is improved, as the intrinsic contact angle of the CPES copolymers decreases and the surface roughness of the supports increases (in Table 3).


(7) $${\mathrm{Cos}\theta }_{A}=\left(1\;-\;f\right){\psi \cos\theta }_{I}-\;f$$

**Figure 4:**
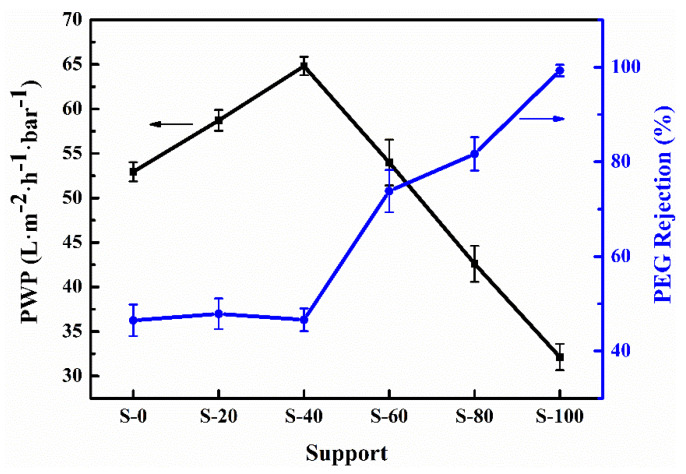
PWP and rejection rate of the supports.

The correlation between the pure water permeability (PWP) and rejection rate (R) of the supports is plotted in Figure 4. The error bars indicate the standard deviation of the average values obtained from five test runs (details about the data can be seen in Tables S5 and S6 (Supplementary Materials)). With the increasing MR of carboxyl units in the CPES copolymers, the PWP of S-0~S-40 increases from 52.95 L·m^−2^·h^−1^·bar^−1^ to 64.82 L·m^−2^·h^−1^·bar^−1^ without impacting their rejection rates (46.49~47.86%). Further, the PWP of S-60~S-100 decreases substantially from 53.99 L·m^−2^·h^−1^·bar^−1^ to 32.12 L·m^−2^·h^−1^·bar^−1^, whereas their rejection rates increase from 73.83% to 99.30%. That is, a typical trade-off between permeability and selectivity is shown in the separation performances of S-60~S-100. In a previous study [29], Kim and Lee found that with the increase in the ratio of PEG (additive) to NMP (solvent) corresponding to a fixed PSF concentration (15 wt%) and other process conditions, the surface pore sizes of the resulting membranes became bigger, and their top layers became more porous. Thus, by increasing the ratio of PEG to NMP, the prepared membranes had a higher water flux but a lower rejection rate. However, as noted earlier in our present paper, the PEG additive with good water solubility can be easily removed from the membranes, which is clearly different from our chemical modification of the support materials (i.e., CPES). Here, the swelling of the hydrophilic support materials can cause pore shrinkage. Consequently, when the MR of carboxyl units in the CPES copolymers is lower (i.e., S-0~S-40), the surface porous structure of the supports does not change due to the slight swelling, thereby leading to no apparent change in selectivity. Meanwhile, the increase in carboxyl content in the CPES copolymers should be a major contributor to the improvement in permeabilities. Further, the excessive swelling of the supports (i.e., S-60~S-100) plays a decisive role in the separation performances. Similar behavior was also observed in a recent study by Cho et al. [21].

### 3.2. Effects of Surface Hydrophilicity of Supports on the Characteristics and Permselectivity of PA-TFC NF Membranes

To investigate the interrelation between the surface hydrophilicity of the supports and the properties of the NF membranes, three representative supports were carefully chosen as the sublayers in the following IP reaction:

(1) S-0 is featured with a relatively hydrophobic surface (CA: 90.43°).
(2) S-40 possesses an increasing surface hydrophilicity (CA: 87.56°).
(3) S-100 has a more hydrophilic surface (CA: 82.12°).

What is remarkable about the selected range of hydrophilicity (82.12°~90.43°) is that a further raising of hydrophilicity will cause excessive swelling, leading to a nonporous membrane, a poor membrane compressive property etc.

**Figure 5:**
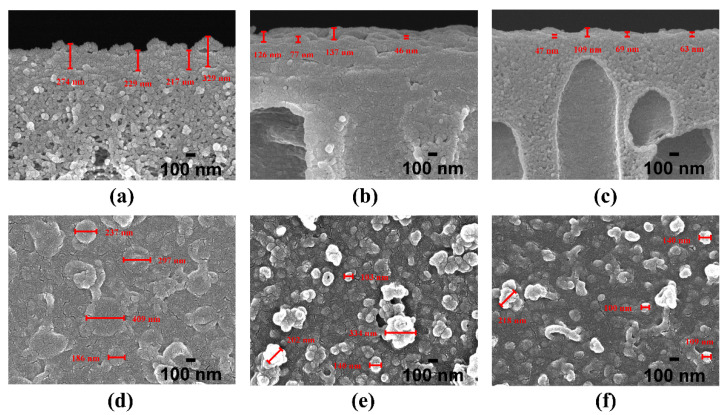
Cross-section and surface FESEM images of PA layer in NF membranes: (**a**,**d**) correspond to N-0; (**b**,**e**) N-40; (**c**,**f**) N-100. The apparent thickness and protuberance size of PA layers are separately labeled in each cross-section and surface image.

The cross-section and surface micrographs of the PA layer in the NF membranes are presented in Figure 5. By visual inspection, it is evident that PA layers with an irregular shape were successfully synthesized on the top of the supports. From the cross-section morphologies in Figure 5, the apparent thickness (δ_N_) of the PA layers is visually characterized by the length of the red drawn lines using Image J software. As listed in Table 4, with the increasing surface hydrophilicity of the supports, the apparent thickness of the PA layers markedly reduces from 262 nm for N-0 to 72 nm for N-100. A comparable observation regarding the decrease in the apparent thickness of the PA layers was also reported in recent literature [30]. Previous studies have illustrated that the apparent thickness of PA layers depends strongly on the reactive monomer concentrations and the ratio of reactant concentrations, which play a big part in the amine molecules penetrating deeper into the polymerization zone [9,31,32,33]. For instance, following a systematic study about the effects of PIP concentrations (0.5~5.0% (*w*/*v*)) on the formation of PA layers corresponding to a fixed TMC concentration (0.1% (*w*/*v*)) and other reaction conditions by Saha et al. [9], it was concluded that at lower PIP concentrations (≤1.5% (*w*/*v*)), the apparent thickness of PA layers increased with the increase in PIP concentrations. With further increasing PIP concentrations (1.5~5.0% (*w*/*v*)), the apparent thickness of the PA layers remained nearly unchanged, but the density of the PA layers increased. Additionally, through a molecular simulation of PA synthesis [33], Nadler and Srebnik indicated that a higher concentration of PIP could give rise to the increase in the density of the PA layer due to the increase in PIP monomers that could aggregate in the rich region of the amino end group in the formed PA layer. Thus, as for the variation in the apparent thickness of the PA layers in our study, one possible reason is that the supports with a higher surface hydrophilicity (e.g., S-40 and S-100) can form a stronger interaction not only with water molecules but also with PIP monomers, which suppresses the diffusion of PIP monomers in the aqueous phase (that is, the actual PIP concentration involved in the IP reaction is lower than its formulated concentration). As such, the increase in the surface hydrophilicity of the supports is conducive to the preparation of a thinner PA layer.

Besides, the obvious differences in the aspects of number and size (λ_N_, the randomly selected protuberances are measured on each surface FESEM image of PA layers in Figure 5, and then the measurements are averaged as listed in Table 4) of protuberances are evident from the surface morphology of the PA layers in Figure 5. The PA layer surface of N-0 clearly exhibits relatively fewer but larger protuberances (λ_N_: 282 nm). Increasing with the surface hydrophilicity of the supports, the PA layer surface of N-40 appears to have more small nodular features (λ_N_: 195 nm). Eventually, many even smaller nodules (λ_N_: 142 nm) are observed on the PA layer surface of N-100 with the further increase in the surface hydrophilicity of the supports. The surface morphological variation of the PA layers can be explained as follows. Generally, interfacial polymerization initiates immediately after the contact of the aqueous phase and the organic phase, leading to the formation of PA films near the organic phase side of the aqueous–organic interface [33]. Based on an in situ-visualized technology and a slow IP reaction between N-methyldiethanolamine (MEDA) and trimesoyl chloride (TMC), Yuan et al. [34] observed that with prolonging the reaction time, the newly formed large bubble-like features would cover the small ones previously formed. Moreover, when the thickness of the interfacial layer increased to the maximum, no new bubble-like features would be formed. Similarly, in our present study, due to the fact that the actual PIP concentrations involved in the IP process decrease with the increase in the surface hydrophilicity of the supports from S-0 to S-100, the corresponding extent of IP reactions may gradually decline. Thus, it appears to be that during the initial stage of the IP reaction, many small nodules appear on the PA layer surface of N-100. As the reaction proceeds, some newly formed large nodular structures of N-40 cover the small nodules of N-100, and, finally, a small amount of the largest protuberances cover the PA layer surface of N-40.

**Figure 6:**
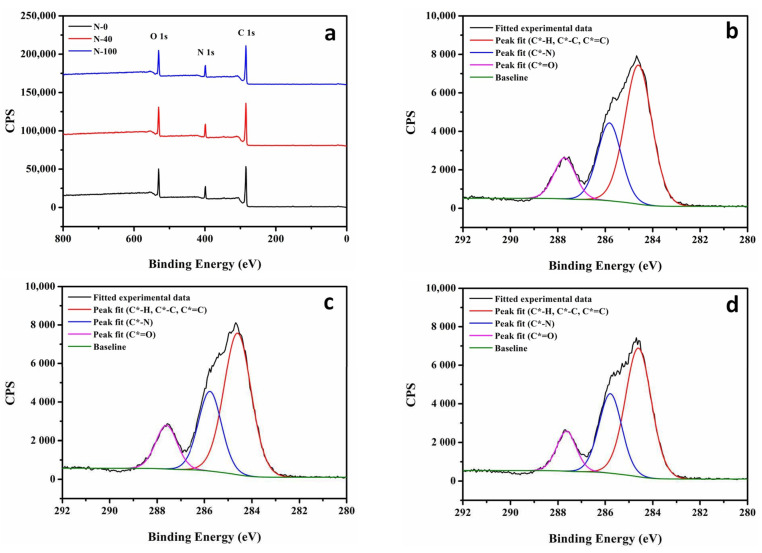
(**a**). XPS survey spectra of PA layer in NF membranes. Deconvolution of C1s core level XPS spectra concerning the PA layer of NF membranes: (**b**). N-0, (**c**). N-40 and (**d**). N-100.

The surface element information of the PA layer in the NF membranes is assessed by X-ray photoelectron spectroscopy (XPS), and the spectrum analyses and statistical results are provided in Figure 6 and Table 5, respectively. Theoretically, from the wide spectra result of XPS, the surface cross-linking degree of the PA layer can be characterized by the ratio of O/N, which is always between 1 and 2, due to the fact that a PA-TMC membrane may possess the cross-linked and linear structures at the same time [4]. More specifically, an O/N ratio of 1 suggests that the PA layer surface is fully cross-linked, while 2 indicates that the PA layer surface is completely linear. In this study, the ratio of O/N decreases from the highest to lowest according to N-0 (1.44) > N-100 (1.41) > N-40 (1.37), validating that the PA layer surface of N-40 is denser than that of N-0 and N-100.

Further, the C1s core level XPS spectra of different NF membranes (Figure 6a) are separately deconvoluted into three peaks representing various carbon functional groups as illustrated in Figure 6b–d. From the results summarized in Table 5, the relative concentration of carbon atoms in C*=O (287.64 eV, representing the sum relative concentration of carbon atoms in N-C*=O and O-C*=O) is in the order of N-40 (14.15%) > N-0 (13.72%) > N-100 (13.38%). Generally, a cross-linked structure of the PA layer is synthesized upon the formation of amide bonds through the interfacial polymerization reaction [7]. However, part of the TMC molecules, which do not undergo cross-linking reactions completely, are prone to hydrolysis to form carboxyl groups when exposed to water [35,36,37,38]. A high content of amide bonds or carboxyl groups is usually believed to help improve the hydrophilicity of materials [38,39]. Thus, the surface material of the PA layer in N-40 with a high relative concentration of carbon atoms in C*=O seems to be more hydrophilic.

However, as presented in Table 4, the surface hydrophilic result (reported in terms of the contact angle (CA)) of the PA layer in the NF membranes decreases from 38.41° for N-0 to 18.94° for N-100 with the increase in the surface hydrophilicity of the supports. As aforementioned in Equation (7) [28], the intrinsic contact angle (θ_I_) of materials on the surface of PA layers and the surface porosity coefficient (f) and the surface roughness coefficient (ψ) of PA layers jointly determine the apparent contact angle (θ_A_) of PA layers. Obviously, both the surface porosity factor (represented in the surface cross-linking degree of the PA layers, i.e., the ratio of O/N in Table 5) and the surface roughness factor (reported in terms of root mean square (RMS) roughness listed in Table 4) serve a secondary role in impacting the surface hydrophilicity of PA layers. For example, as the polyamide is hydrophilic (θ_I_ < 90°), the PA layer of N-40 with the lowest surface porosity and the highest surface roughness among all the NF membranes does not show a relatively good hydrophilicity. So, we speculate that as the surface hydrophilicity of the supports increases, the improved hydrophilicity of the materials on the surface of PA layers (i.e., the θ_I_ is reduced) would play a dominant role in the surface hydrophilicity of PA layers. As discussed earlier though, the relative concentration of carbon atoms in C*=O on the surface of the PA layers is inconsistent with the surface hydrophilic result of the PA layers. This may be due to the fact that the concentration result of the elements obtained by XPS is a relative value expressed in percentage. Then, it is highly possible that the formation of the PA layer surface prepared on the support with a better hydrophilicity would undergo interfacial polymerization more times, causing a large absolute number of hydrophilic functional groups.

**Figure 7:**
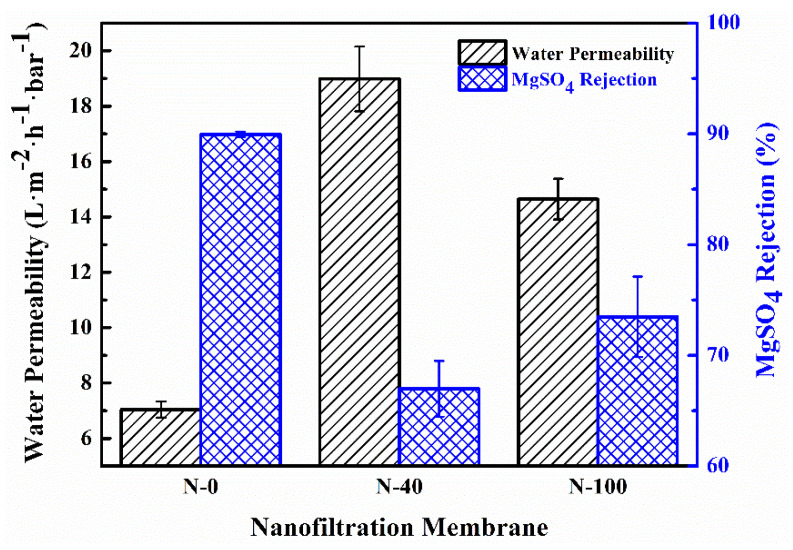
Separation performances of NF membranes.

The surface hydrophilicity of the supports has a critical influence on the separation performances of the NF membranes as shown in Figure 7. The error bars indicate the standard deviation of average values obtained from three test runs (details about the data can be seen in Tables S7 and S8 (Supplementary Materials)). As the surface hydrophilicity of the supports increases, the water permeability of the NF membranes increases dramatically from 7.04 L·m^−2^·h^−1^·bar^−1^ (N-0) to 19.25 L·m^−2^·h^−1^·bar^−1^ (N-40), while the MgSO_4_ rejections decrease from 89.94% to 66.98%. Further, the water permeability of N-100 decreases to 14.64 L·m^−2^·h^−1^·bar^−1^, and the MgSO_4_ rejection increases to 73.48%.

For a long time, many studies focusing on the transport through osmotic membranes related the permselectivity of the TFC membranes to their distinct physicochemical properties when the solute materials were fixed [40]. Some research was inclined to attribute the high water permeability to a thin PA layer, arising from less PA films synthesized in the surface pore of the support. Nevertheless, a recent mathematical model [7] and direct microscopic observations [41,42] concerning the PA layer have confirmed that the IP reaction occurs mainly on the organic phase side, and, then, the penetration of PA films into the support should be almost negligible. Therefore, according to Table 4, the apparent thickness of the PA layer in N-100 is thinner than that in N-40, meaning that the water permeability of the former is supposed to be higher than that of the latter. However, this is not consistent with the fact that the water permeability of N-100 is lower than that of N-40, indicating that the water permeabilities under discussion are not obviously influenced by the thickness of the PA layers. In this regard, Ghosh et al. [13] also suggested that the thickness of PA layers in TFC membranes was not intrinsically related to water permeability.

Furthermore, noting the typical trade-off relationship between the permeability and selectivity of NF membranes, it is suggested that the compactness (represented in terms of cross-linking degree) of PA layers should be the major cause for the separation performances. However, our conjecture is inconsistent with the surface cross-linking degree of PA layers (i.e., the ratio of O/N shown in Table 5). For instance, N-0 with the lowest surface cross-linking degree has the lowest water permeability among all the NF membranes. This is probably due to the shallow detection depth of the XPS measurement (<10 nm) [7,43]. So, it is unreasonable to predict the actual structural properties of entire PA layers just from their surface cross-linking degree results.

We noted that previous theoretical and experimental studies [7,43] demonstrated that there was a continuous dense core (or a base film) at the bottom of PA layers, which was usually embedded in a loose film periphery. In this study, it is more likely that the dense core of the PA layers acts as an actual selective barrier responsible for the separation performances. Following the logic developed above, it appears reasonable to conclude that the cross-linking degree of the dense core in the PA layers is in the following order: N-0 > N-100 > N-40.

**Figure 8:**
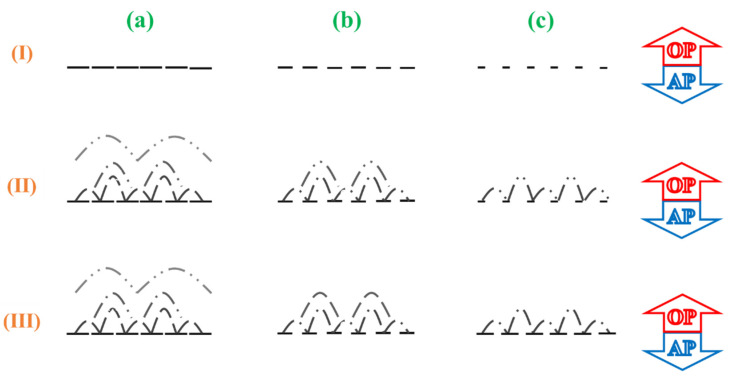
Schematic representation for the formation of PA layers in three representative NF membranes ((**a**) N-0, (**b**) N-40 and (**c**) N-100) through the (**I**) formation of inner nascent films; (**II**) formation of outer loose layers; (**III**) densification of PA layers. The solution environments on both sides of the interface are “oil phase” (OP) and “aqueous phase” (AP). In phase (**II**) and (**III**), the density of loose layers is presented with lines as follows: “
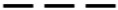
” refers to be dense; “
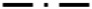
” medium; “
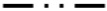
” loose.

Overall, in Figure 8, a conceptual scenario based on the kinetics of film formation via interfacial polymerization [7] is proposed to depict the possible formation mechanism of PA layers in NF membranes (e.g., N-0, N-40 and N-100) over corresponding supports with different surface hydrophilicity. After the fast formation of nascent films (or dense barriers) (see Figure 8(I)), the interfacial polymerization begins to shift to a diffusion-controlled process: during the initial stage arising from being dominated by the PIP monomers diffusing toward the outside of the nascent films, the loose layers with bubble-like features start to grow outward (see Figure 8(II)); yet, in the subsequent stage, stemming from being led by the diffusion of TMC molecules toward the nascent films, the density of the PA layers is improved from the surface to the inside (see Figure 8(III)). This process can be reformulated with more detail as follows.

In the first phase, immediately after the aqueous phase contacts with the organic phase, interfacial polymerization initiates, leading to the formation of nascent films [6,7]. With the increase in the surface hydrophilicity of the supports from S-0 to S-100, the actual concentration of PIP in the aqueous phase participating in the polymerization decreases as the order of S-0 < S-40 < S-100, and, thereby, the defects of the corresponding nascent films (i.e., the spacing between dashed lines in Figure 8(I)) increase gradually. In the second phase, the nascent films of each NF membrane divide the polymerization zone into two regions, and each region is rich in one type of monomer and/or its reactive functional groups [33]. With the PIP monomers diffusing through the nascent films and then becoming consumed by the TMC molecules or its unreacted functional groups persistently, the loose layers grow in an upward direction. Specifically, the apparent thickness of the PA layers decreases from N-0 to N-100 with the increasing surface hydrophilicity of the supports. Besides, as contended by Yuan et al. [34], the big bubble-like features that are newly formed will cover the small ones previously formed. Moreover, in the third stage, the thickness of the PA layers remains almost unchanged, while the densities change from the surface to the inside [33]. In detail, since the paths for PIP monomers to diffuse through the dense nascent films and the thick loose layer of N-0 are tortuous, the polymerization is almost slowed down abruptly, and a density change in the PA layer in N-0 is not evident. By contrast, it becomes relatively easy for PIP monomers to diffuse and then become consumed in the case of N-40, leading to the significant increase in the surface density of the PA layer. However, there is no significant change in the nascent films, and, further, in the case of N-100, it is increasingly easy for the diffusion of PIP monomers. However, compared to the case of N-40, due to the fact that the actual concentration of PIP in the aqueous phase participating in the polymerization decreases, the TMC molecules are more likely to go deep into the PA layer for a reaction. Accordingly, the density of the PA layer in N-100 increases from the surface to the bottom. It is worth noting that the surface hydrophilicity of PA layers is associated with their material hydrophilicity, that is, the times of IP reaction.

## 4. Conclusions

In this study, by adopting a series of carboxylated PES copolymers featuring an increasing molar ratio of carboxyl units, supports with different surface hydrophilicities were fabricated via the NIPS method. For the PA-TFC NF membranes, due to the fact that the concentration of PIP in the aqueous phase actually involved in the reaction decreases with the increase in the surface hydrophilicity of supports, thin PA layers with a low cross-linking degree of dense cores were formed on the relatively hydrophilic supports. On the contrary, a thick PA layer with a high cross-linking degree of dense core was synthesized on the relatively hydrophobic support. So, the NF membrane prepared on the hydrophobic support has a lower water permeability and a higher salt rejection than the ones prepared on the hydrophilic supports. Our direct experimental evidence has further revealed the impacts of the surface hydrophilicity of supports on the characteristics and permselectivity of the resulting PA-TFC NF membranes. In the future, this paper is bound to assist us in selecting suitable hydrophilic supports for achieving the desired NF membrane performances.

## Figures and Tables

**Table 1:** Nomenclature of CPES copolymers, supports and NF membranes.

CPES Copolymer	Support	NF Membrane
CPES-0 ^a^	S-0 ^b^	N-0 ^c^
CPES-20	S-20	/
CPES-40	S-40	N-40
CPES-60	S-60	/
CPES-80	S-80	/
CPES-100	S-100	N-100

^a^ means that the MR of carboxyl units in CPES copolymer is 0; ^b^ means that the support was prepared by CPES-0; ^c^ shows that the NF membrane was prepared on S-0. By analogy, the meaning of other symbols can be reached.

**Table 2:** Test conditions for the water permeability and rejection rate of NF membranes.

Membrane	Test solution	Concentration (mgL^−1^)	pH	Temperature (°C)	Pressure (MPa)	MSV ^a^ (ms^−1^)
NF	MgSO_4_	2000 ± 20	7.5 ± 0.5	25.0 ± 0.2	0.60 ± 0.02	≥0.45

^a^ refers to the “surface flow velocity” (MSV) of test solutions over the membranes.

**Table 3:** Pore size (d), thickness (δ_S_) and root mean square (RMS) roughness and contact angle (CA) of the supports.

Support	S-0	S-20	S-40	S-60	S-80	S-100
d (nm)	9	9	9	9	8	4
δ_S_ (μm)	48 ± 1	50 ± 1	48 ± 1	49 ± 1	48 ± 1	48 ± 1
RMS (nm)	18.3 ± 1.2	22.7 ± 2.0	27.5 ± 2.1	36.6 ± 2.2	41.3 ± 2.6	49.4 ± 1.4
CA (deg.)	90.43 ± 0.86	87.93 ± 0.44	87.56 ± 0.22	87.11 ± 0.37	86.43 ± 0.92	82.12 ± 0.65

**Table 4:** Apparent thickness (δ_N_), protuberance size (λ_N_) and root mean square (RMS) roughness and contact angle (CA) of PA layer in NF membranes.

NF Membrane	N-0	N-40	N-100
δ_N_ (nm)	262 ± 51	96 ± 43	72 ± 26
λ_N_ (nm)	282 ± 96	195 ± 102	142 ± 54
CA (deg.)	38.41 ± 0.71	33.92 ± 1.02	18.94 ± 0.75
RMS (nm)	41.2 ± 1.7	78.5 ± 2.4	45.0 ± 0.4

**Table 5:** XPS scan results for the PA layer surface in NF membranes.

NF Membrane	O 1s (%)	N 1s (%)	C 1s (%)	O/N	C*=O (%)
N-0	14.01 ± 0.53	9.78 ± 0.82	76.21 ± 0.22	1.44 ± 0.07	13.72 ± 0.61
N-40	13.95 ± 0.59	10.17 ± 0.74	75.87 ± 0.24	1.37 ± 0.04	14.15 ± 0.65
N-100	14.27 ± 0.69	10.14 ± 0.86	75.59 ± 0.21	1.41 ± 0.06	13.38 ± 0.72

## Data Availability

The data presented in this study are available on request from the corresponding author.

## Supplementary Materials

The following are available online at https://www.mdpi.com/article/10.3390/nano11102470/s1, Table S1: Viscosity of the casting solutions, Figure S1: MWCO of the supports, Table S2: MWCO of the supports, Table S3: Contact angle (CA) of the supports, Table S4: ICA of CPES copolymers, Table S5: PWP of the supports, Table S6: Rejection rate (R) of the supports, Table S7: Water permeability (A) of the NF membranes, Table S8: Salt rejection (R) of the NF membranes.

## References

[1] Loo S.L., Fane A.G., Krantz W.B., Lim T.T. (2012). Emergency water supply: A review of potential technologies and selection criteria. Water Res..

[2] Elimelech M., Phillip W.A. (2011). The future of seawater desalination energy, technology, and the environment. Science.

[3] Logan B.E., Elimelech M. (2012). Membrane-based processes for sustainable power generation using water. Nature.

[4] Karan S., Jiang Z., Livingston A.G. (2015). Sub-10 nm polyamide nanofilms with ultrafast solvent transport for molecular separation. Science.

[5] Li D., Yan Y., Wang H. (2016). Recent advances in polymer and polymer composite membranes for reverse and forward osmosis processes. Prog. Polym. Sci..

[6] Petersen R.J. (1993). Composite reverse osmosis and nanofiltration membranes. J. Membr. Sci..

[7] Freger V. (2005). Kinetics of film formation by interfacial polycondensation. Langmuir.

[8] Yu S., Liu M., Liu X., Gao C. (2009). Performance enhancement in interfacially synthesized thin-film composite polyamide-urethane reverse osmosis membrane for seawater desalination. J. Membr. Sci..

[9] Saha N.K., Joshi S.V. (2009). Performance evaluation of thin film composite polyamide nanofiltration membrane with variation in monomer type. J. Membr. Sci..

[10] Kim I.C., Jeong B.R., Kim S.J., Lee K.H. (2013). Preparation of high flux thin film composite polyamide membrane: The effect of alkyl phosphate additives during interfacial polymerization. Desalination.

[11] Xiang J., Xie Z., Hoang M., Zhang K. (2013). Effect of amine salt surfactants on the performance of thin film composite poly(piperazine-amide) nanofiltration membranes. Desalination.

[12] Zou H., Jin Y., Yang J., Dai H., Yu X., Xu J. (2010). Synthesis and characterization of thin film composite reverse osmosis membranes via novel interfacial polymerization approach. Sep. Purif. Technol..

[13] Ghosh A.K., Jeong B.H., Huang X., Hoek E.M.V. (2008). Impacts of reaction and curing conditions on polyamide composite reverse osmosis membrane properties. J. Membr. Sci..

[14] Singh P.S., Joshi S.V., Trivedi J.J., Devmurari C.V., Rao A.P., Ghosh P.K. (2006). Probing the structural variations of thin film composite RO membranes obtained by coating polyamide over polysulfone membranes of different pore dimensions. J. Membr. Sci..

[15] Huang L., McCutcheon J.R. (2015). Impact of support layer pore size on performance of thin film composite membranes for forward osmosis. J. Membr. Sci..

[16] Misdan N., Lau W.J., Ismail A.F., Matsuura T. (2013). Formation of thin film composite nanofiltration membrane: Effect of polysulfone substrate characteristics. Desalination.

[17] Li X., Li Q., Fang W., Wang R., Krantz W.B. (2019). Effects of the support on the characteristics and permselectivity of thin film composite membranes. J. Membr. Sci..

[18] Maruf S.H., Greenberg A.R., Ding Y. (2016). Influence of substrate processing and interfacial polymerization conditions on the surface topography and permselective properties of surface-patterned thin-film composite membranes. J. Membr. Sci..

[19] Ghosh A.K., Hoek E.M.V. (2009). Impacts of support membrane structure and chemistry on polyamide-polysulfone interfacial composite membranes. J. Membr. Sci..

[20] Kim H.I., Kim S.S. (2006). Plasma treatment of polypropylene and polysulfone supports for thin film composite reverse osmosis membrane. J. Membr. Sci..

[21] Cho Y.H., Han J., Han S., Guiver M.D., Park H.B. (2013). Polyamide thin-film composite membranes based on carboxylated polysulfone microporous support membranes for forward osmosis. J. Membr. Sci..

[22] Shi M., Yan W., Zhou Y., Wang Z., Liu L., Zhao S., Ji Y., Wang J., Gao C., Zhang P. (2020). Combining tannic acid-modified support and a green co-solvent for high performance reverse osmosis membranes. J. Membr. Sci..

[23] Yuan S., Li J., Zhu J., Volodine A., Li J., Zhang G., Van Puyvelde P., Van der Bruggen B. (2018). Hydrophilic nanofiltration membranes with reduced humic acid fouling fabricated from copolymers designed by introducing carboxyl groups in the pendant benzene ring. J. Membr. Sci..

[24] Doan V., Ko1ppe R., Kasai P.H. (1997). Dimerization of Carboxylic Acids and Salts∶An IR Study in Perfluoropolyether Media. J. Am. Chem. Soc..

[25] Lentsch S., Aimar P., Orozco J.L. (1993). Separation albumin-PEG: Transmission of PEG through ultrafiltration membranes. Biotechnol. Bioeng..

[26] Kesting R.E. (1990). The four tiers of structure in integrally skinned phase inversion membranes and their relevance to the various separation regimes. J. Appl. Polym. Sci..

[27] Zhenxin Z., Matsuura T. (1991). Discussions on the formation mechanism of surface pores in reverse osmosis, ultrafiltration, and microfiltration membranes prepared by phase inversion process. J. Colloid Interface Sci..

[28] Cassie A.B.D., Baxter S. (1944). Wettability of porous surfaces. Trans. Faraday Soc..

[29] Kim J.-H., Lee K.-H. (1998). Effect of PEG additive on membrane formation by phase inversion. J. Membr. Sci..

[30] Shi M., Wang Z., Zhao S., Wang J., Zhang P., Cao X. (2018). A novel pathway for high performance RO membrane: Preparing active layer with decreased thickness and enhanced compactness by incorporating tannic acid into the support. J. Membr. Sci..

[31] Chai G., Krantz W.B. (1994). Formation and characterization of polyamide membranes. J. Membr. Sci..

[32] Freger V., Srebnik S. (2003). Mathematical model of charge and density distributions in interfacial polymerization of thin films. J. Appl. Polym. Sci..

[33] Nadler R., Srebnik S. (2008). Molecular simulation of polyamide synthesis by interfacial polymerization. J. Membr. Sci..

[34] Yuan F., Wang Z., Yu X., Wei Z., Li S., Wang J., Wang S. (2012). Visualization of the formation of interfacially polymerized film by an optical contact angle measuring device. J. Phys. Chem. C.

[35] Tiraferri A., Elimelech M. (2012). Direct quantification of negatively charged functional groups on membrane surfaces. J. Membr. Sci..

[36] Freger V. (2003). Nanoscale heterogeneity of polyamide membranes formed by interfacial polymerization. Langmuir.

[37] Tang C.Y., Kwon Y.-N., Leckie J.O. (2009). Effect of membrane chemistry and coating layer on physiochemical properties of thin film composite polyamide RO and NF membranes I. FTIR and XPS characterization of polyamide and coating layer chemistry. Desalination.

[38] Yip N.Y., Tiraferri A., Phillip W.A., Schiffman J.D., Elimelech M. (2010). High performance thin-film composite forward osmosis membrane. Environ. Sci. Technol..

[39] Lee A., Elam J.W., Darling S.B. (2016). Membrane materials for water purification: Design, development, and application. Enviorn. Sci. Water Res..

[40] Wang J., Dlamini D.S., Mishra A.K., Pendergast M.T.M., Wong M.C.Y., Mamba B.B., Freger V., Verliefde A.R.D., Hoek E.M.V. (2014). A critical review of transport through osmotic membranes. J. Membr. Sci..

[41] Zhang Y., Benes N.E., Lammertink R.G. (2015). Visualization and characterization of interfacial polymerization layer formation. Lab Chip.

[42] Wang J., Xu R., Yang F., Kang J., Cao Y., Xiang M. (2018). Probing influences of support layer on the morphology of polyamide selective layer of thin film composite membrane. J. Membr. Sci..

[43] Wong M.C.Y., Lin L., Coronell O., Hoek E.M.V., Ramon G.Z. (2016). Impact of liquid-filled voids within the active layer on transport through thin-film composite membranes. J. Membr. Sci..

