# Supplementary material for: Impacts of Surface Hydrophilicity of Carboxylated Polyethersulfone Supports on the Characteristics and Permselectivity of PA-TFC Nanofiltration Membranes
Source: Nanomaterials (Basel). 2021 Sep 22;11(10):2470. doi: 10.3390/nano11102470 (PMC8541453; doi:10.3390/nano11102470)
Supplement: Supplementary file 1 [file nanomaterials-11-02470-s001.zip › nanomaterials-1347112-supplementary.pdf]

Supplementary information

Impacts of Surface Hydrophilicity of Carboxylated Polyethersulfone Supports on the Characteristics and Permselectivity of PA-TFC Nanofiltration Membranes

Yingfu Lian [1], Gang Zhang [2,3*], Xiaojun Wang [2] and Jie Yang [2,3*]

[1] College of Polymer Materials Science and Engineering, Sichuan University, Chengdu 610064, China
[2] Institute of Materials Science and Technology, Analytical & Testing Center, Sichuan University, Chengdu, 610064, China
[3] State Key Laboratory of Polymer Materials Engineering of China, Sichuan University, Chengdu 610064, China
* Correspondence: Correspondence: gangzhang@scu.edu.cn, ppsf@scu.edu.cn

1. Viscosity of Casting Solutions for Preparing the Supports

The "viscosity of casting solutions" (η) was measured with a NDJ-8S digital rotational rheometer (Shanghai YoYi Instrument Co. Ltd., Shanghai, China). After the temperature of hollow cylinder was controlled at 15 °C (same to the temperature of coagulating bath) through a circulating water system, one of casting solutions was poured into the cylinder. Then sufficient time was allowed for the test unit to attain thermal equilibrium. And ultimately the viscosity was determined by using a 2# rotor and in the rotor speed of 6 RPM (margin viscosity test: 5000 mPa·s).

Table S1. Viscosity of the casting solutions.

Support	S-0	S-20	S-40	S-60	S-80	S-100
η (mPa·s)	1600±10	2247±6	2690±10	3167±6	3803±6	4667±6

2. Molecular Weight Cut Off (MWCO) of Supports

MWCO refers to the molecular weight that 90% rejection is reached through the selective experiments with reference materials (e.g., PEG-6,000, 10,000, 20,000, 35,000 and 40,000).

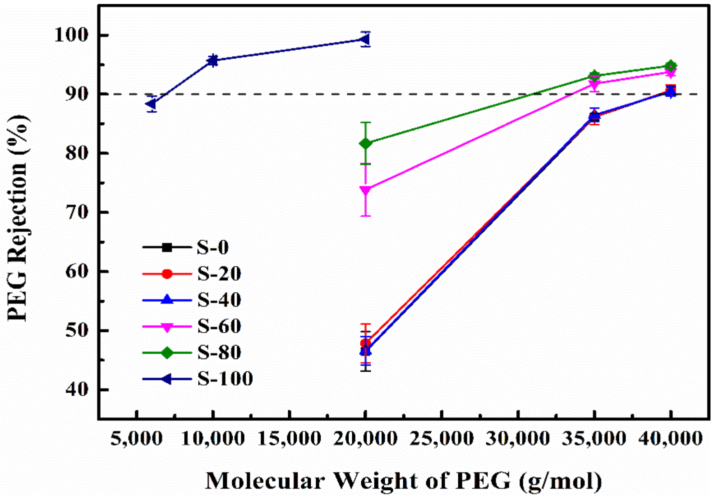

Figure S1. MWCO of the supports.

Table S2. MWCO of the supports

Support	S-0	S-20	S-40	S-60	S-80	S-100
**MWCO (g/mol)**	39,250	39,250	39,250	33,275	30,750	6,875

3. Contact Angle (CA) of the Supports

Table S3. Contact angle (CA) of the supports.

Support		S-0	S-20	S-40	S-60	S-80	S-100
**CA (deg.)**	1	91.46	88.53	87.59	86.72	85.07	82.96
	2	90.88	88.1	87.72	87.58	85.95	82.62
	3	90.65	87.59	87.22	86.88	86.86	81.95
	4	89.88	88.02	87.78	86.94	86.92	81.64
	5	89.26	87.41	87.47	87.41	87.35	81.42
	Mean	90.43	87.93	87.56	87.11	86.43	82.12
	Standard Deviation	0.86	0.44	0.22	0.37	0.92	0.65

4. Intrinsic Contact Angle (ICA) of CPES Copolymers

For measuring the ICA of CPES copolymers, the making process of dense membranes is described briefly below. First, a certain amount of casting solutions (15 wt%) were prepared by dissolving CPES copolymers with different MR of carboxyl units in NMP separately. After coating glass slides one by one with the fully dissolved casting solutions, they were together put into a vacuum oven acclimated to 80 °C for 8 h to volatilize the solvent. Subsequently the temperature of oven was raised up to 100 °C for another 8 h. Eventually the membranes were dried at 120 °C and -0.09 MPa vacuum degrees for another 8 h to ensure that the solvent was removed completely.

Table S4. ICA of CPES copolymers

CPES	CPES-0	CPES-20	CPES-40	CPES-60	CPES-80	CPES-100
**ICA (deg.)**	77.83±0.5	72.42±0.4	70.07±0.4	68.37±0.4	66.53±0.5	64.61±0.3

5. Pure Water Permeability (PWP) and Rejection Rate (R) of the Supports

Table S5. PWP of the supports.

Support		S-0	S-20	S-40	S-60	S-80	S-100
**PWP ($L\cdot m^{-2}\cdot h^{-1}\cdot bar^{-1}$)**	1	53.63	59.78	64.36	51.42	42.23	33.85
	2	53.28	57.3	65.73	55.89	40.47	30.79
	3	52.35	60.09	64.59	51.1	40.95	33.45
	4	51.42	58.08	63.45	54.93	44.76	30.69
	5	54.08	58.37	65.95	56.62	44.65	31.8
	Mean	52.95	58.72	64.82	53.99	42.61	32.12
	Standard Deviation	1.07	1.18	1.03	2.57	2.02	1.47

Table S6. Rejection rate (R) of the supports.

Support		S-0	S-20	S-40	S-60	S-80	S-100
**R (%)**	1	43.41	48.23	44.2	70.17	78.12	100
	2	42.92	44.15	49.06	69.28	85.27	97.18
	3	46.65	44.92	48.18	72.64	84.14	100
	4	49.08	50.83	47.66	78.37	83.16	100
	5	50.38	51.18	43.83	78.67	77.64	99.31

Mean	46.49	47.86	46.59	73.83	81.67	99.30
Standard Deviation	3.32	3.26	2.40	4.46	3.54	1.22

6. Water Permeability and Salt Rejection of the NF Membranes

Table S7. Water permeability (A) of the NF membranes.

NF Membrane		N-0	N-40	N-100
**A ($L \cdot m^{-2} \cdot h^{-1} \cdot bar^{-1}$)**	1	7.05	18.54	15.26
	2	6.75	17.29	13.83
	3	7.33	21.91	14.84
	Mean	7.04	19.25	14.64
	Standard Deviation	0.29	2.39	0.74

Table S8. Salt rejection (R) of the NF membranes.

NF Membrane		N-0	N-40	N-100
**R (%)**	1	90.23	67.46	70.87
	2	89.87	69.23	77.64
	3	89.73	64.26	71.93
	Mean	89.94	66.98	73.48
	Standard Deviation	0.26	2.52	3.64
